# A Three-Dimensional Model of Women’s Empowerment: Implications in the Field of Microfinance and Future Directions

**DOI:** 10.3389/fpsyg.2017.01678

**Published:** 2017-09-28

**Authors:** Marloes A. Huis, Nina Hansen, Sabine Otten, Robert Lensink

**Affiliations:** ^1^Department of Social Psychology, University of Groningen, Groningen, Netherlands; ^2^Department of Economics, Econometrics, and Finance, University of Groningen, Groningen, Netherlands; ^3^Development Economics Group, Wageningen University Wageningen, Netherlands

**Keywords:** empowerment, agency, efficacy, gender relations, women, microfinance, culture

## Abstract

Women’s empowerment is an important goal in achieving sustainable development worldwide. Offering access to microfinance services to women is one way to increase women’s empowerment. However, empirical evidence provides mixed results with respect to its effectiveness. We reviewed previous research on the impact of microfinance services on different aspects of women’s empowerment. We propose a Three-Dimensional Model of Women’s Empowerment to integrate previous findings and to gain a deeper understanding of women’s empowerment in the field of microfinance services. This model proposes that women’s empowerment can take place on three distinct dimensions: (1) the micro-level, referring to an individuals’ personal beliefs as well as actions, where personal empowerment can be observed (2) the meso-level, referring to beliefs as well as actions in relation to relevant others, where relational empowerment can be observed and (3) the macro-level, referring to outcomes in the broader, societal context where societal empowerment can be observed. Importantly, we propose that time and culture are important factors that influence women’s empowerment. We suggest that the time lag between an intervention and its evaluation may influence when empowerment effects on the different dimensions occur and that the type of intervention influences the sequence in which the three dimensions can be observed. We suggest that cultures may differ with respect to which components of empowerment are considered indicators of empowerment and how women’s position in society may influence the development of women’s empowerment. We propose that a Three-Dimensional Model of Women’s Empowerment should guide future programs in designing, implementing, and evaluating their interventions. As such our analysis offers two main practical implications. First, based on the model we suggest that future research should differentiate between the three dimensions of women’s empowerment to increase our understanding of women’s empowerment and to facilitate comparisons of results across studies and cultures. Second, we suggest that program designers should specify how an intervention should stimulate which dimension(s) of women’s empowerment. We hope that this model inspires longitudinal and cross-cultural research to examine the development of women’s empowerment on the personal, relational, and societal dimension.

## Introduction

Throughout history and across nations still today, men on average have greater access to power (e.g., Brown, 1991; United Nations Development Programme [UNDP], 2015). The gender power model (Pratto and Walker, 2004; Pratto et al., 2011) suggests that power is gendered. Specifically, men relative to women have greater access to the use of force, greater access to resource control, less social obligations to uphold, and more advantageous cultural ideologies. This gender inequality can be observed in several aspects of daily life such as access to education, job opportunities, and economic resources (United Nations Development Programme [UNDP], 2015). According to a report by the United Nations Educational Scientific and Cultural Organization [UNESCO] (2014), in 2011 only 20% of the low-income nations had achieved gender parity in primary education and 66% of the world’s 774 million illiterate adults were still women. There is consensus that gender equity is an important goal to be achieved (e.g., UN Women, 2011). More precisely, world leaders have agreed on working toward providing women and girls with equal access to various domains of social life (United Nations, n.d.). Diverse interventions have been developed and implemented to strengthen the position of women across the world such as health, educational or financial programs (for an overview see, UN Women, 2016). The concept of empowerment has been developed as a framework and process aimed toward addressing the inequity.

Empowerment is a process, from being unpowered to being empowered. Theorizing of empowerment stresses two main perspectives on this process: namely one more individualistic, namely through women’s individual capacities and free exercise of personal choice (e.g., Kabeer, 1999) and one more collectivistic, namely through collective behavior and the adherence to cultural norms which emphasize collective growth (e.g., Budgeon, 2015, Kurtiş et al., 2016). Microfinance interventions are based on the assumption that participation in the intervention have empowering effects and stimulate individual growth. However, these interventions are often implemented in more traditional collectivistic cultures. Thus, it is crucial to conduct cultural sensitive research to avoid cultural biases and understand empowerment outcomes in different cultural contexts.

We provide a framework in which we propose that women’s empowerment can be differentiated in three different dimensions, namely personal, relational (with respect to relevant others such as spouse, family, and community), and societal (at the larger social context) empowerment. We conducted our analysis in the field microfinance services as it offers a large body of empirical studies based on literature from different disciplines, mainly psychology, developmental economics, and sociology, in three steps. First, based on the reviewed literature we define women’s empowerment. Second, we review empirical findings based on the three dimensions of women’s empowerment to illustrate how it has been investigated so far in the context of microfinance services across cultures. Third, we integrate these three dimensions in a Three-Dimensional Model of Women’s Empowerment to improve our understanding of what women’s empowerment entails and how microfinance services may help to increase it.

Offering microfinance services (i.e., microloans, business training, saving programs) is currently one of the most prominent means to reduce poverty and empower the disadvantaged, including women (e.g., Armendáriz and Morduch, 2010; Kulkarni, 2011). The underlying assumption is that market participation will have liberating and empowering effects on women. This neoliberal ideology has been criticized because it neglects to acknowledge local knowledge and practices, and may even reproduce forms of oppression by extending (white) men’s rights to women (e.g., Mohanty, 1995; Roodman, 2011; Bateman and Chang, 2012; Kurtiş et al., 2016). Moreover, systematic reviews on the impact of access to microcredit programs on women’s empowerment have provided inconclusive results (e.g., Van Rooyen et al., 2012; Duvendack et al., 2014; Vaessen et al., 2014). Besides, the controversy of microfinance services, this field of research offers a unique context to conduct our analysis.

## Defining Women’S Empowerment

In the field of development economics women’s empowerment is defined as the process through which women acquire the ability to make strategic life choices in a context where this ability was previously denied to them (Kabeer, 1999). Kabeer (1999) stresses that the ability to exercise individual choice is based on three interrelated elements – *resources, agency*, and *achievements*. Resources refer to material, human, and social expectations and allocations. Agency is the ability or sense of ability to define one’s goals, act upon them, and decide on their own strategic life outcomes. Achievements include a variety of outcomes ranging from improved well-being to achieving equal representation of women in politics. In other words, the underlying assumption is that women’s empowerment is the process of having and using resources in an agentic manner to reach certain achievements (e.g., Kabeer, 1999; Malhotra et al., 2002; Bali Swain and Wallentin, 2009; Khan and Khan, 2016). Similarly, psychological research suggests that empowerment is a process that enables people to act on and improve issues that are important for their individual lives, their communities, and their society (e.g., Bandura, 1986; Page and Czuba, 1999; Maton, 2008; Cattaneo and Chapman, 2010). These definitions stress the expansion of women’s individual capacities and a free exercise of personal choice (see Budgeon, 2015; Kurtiş et al., 2016). However, previous research has highlighted that the act of choosing does not necessarily equate progressive outcomes for women, because women’s individual choices are historically and structurally conditioned (for a debate on choice feminism see Budgeon, 2015). Indeed, this focus on women’s individual liberties and growth is grounded in Western Educated Industrialized Rich Democratic (WEIRD; Henrich et al., 2010) realities and may marginalize the experience of women in different societies (e.g., Carby, 1997; Kurtiş and Adams, 2015). Decolonial feminist psychology stresses the importance of being sensitive to cultural contexts, and gaining insights from (rather than ignoring or devaluing) the experience of women in low income countries (coined majority-world spaces in literature in this field to reflect the majority of humankind inhabiting these societies; Kağitçibaşi, 1995; Kurtiş and Adams, 2015; Kurtiş et al., 2016). In line with this perspective, a recent study (e.g., Dutt et al., 2016) focused on the conception of women’s empowerment through collective rather than individual business ownership, thereby adhering to relevant cultural norms emphasizing collective rather than individual growth (Kurtiş et al., 2016). In the definition of women’s empowerment the collective is also considered. Stromquist (1995) described empowerment as a multifaceted concept including different components ranging from women’s understanding of the causes of their suppression to acting collectively as a group toward social change. The work builds upon the assumption that participation in small groups with a collective agenda is the first step toward women’s empowerment. Individual and collective agency are thus crucial in the development of women’s empowerment (Stromquist, 2015).

Importantly, research so far has studied a variety of very different components of women’s empowerment. Indeed, empirical research has investigated women’s empowerment with measures such as agency, autonomy, capacity for action, self-determination, and self-confidence (e.g., Cheston and Kuhn, 2001; Malhotra et al., 2002; Narayan, 2005; Hansen, 2015). However, all definitions stress that women’s empowerment is a multifaceted concept, which includes different components and assumes that empowerment is a process from being un-empowered to becoming empowered. Combining these views, we propose that *empowerment is a multifaceted process*, which involves individual as well as collective awareness, beliefs, and behavior embedded in the social structure of specific cultural contexts. In the current paper, we next review empirical research in the context of microfinance services to understand (1) which specific components of women’s empowerment are assessed and (2) differentiate those components in personal, relational, and societal empowerment.

## Women’S Empowerment in the Context of Microfinance Services

There is a large body of research on impacts of microfinance services on economic outcomes (for reviews see Banerjee et al., 2015). In the current paper, we have selected research conducted in diverse cultural contexts that has specifically focused on women’s empowerment as an outcome. Previous research reports mixed evidence for the impact of access to microfinance services on women’s empowerment (e.g., Duvendack et al., 2014). The diversity of reported findings may in part be explained by two main methodological differences in the studied interventions. First, microfinance programs greatly differ in their offered services (Armendáriz and Morduch, 2010). Studies report the impacts of a group lending versus individual microcredit programs (e.g., Attanasio et al., 2013)^1^, the impact of training programs differing in content and length (e.g., Kim et al., 2007), or microfinance interventions which may include training, saving activities, and micro loans (e.g., Hansen, 2015). Second, the study designs differ and include results from nationwide demographic survey data (e.g., Banerjee et al., 2015), randomized control trials (e.g., Tarozzi et al., 2015), behavioral games (e.g., Bulte et al., 2016), or semi-structured in-depth interviews (e.g., Sanyal, 2009)^2^. Together, these two main methodological differences make it difficult to systematically compare results and are important to keep in mind (for a review paper see Duvendack et al., 2014).

To integrate the findings of previous research, and to gain a deeper understanding of women’s empowerment in the field of microfinance services, we propose a Three-Dimensional Model of Women’s Empowerment. This model assumes that women’s empowerment can be differentiated at three distinct dimensions: (1) the micro-level dimension, referring to individuals’ personal beliefs as well as actions where personal empowerment can be observed, (2) the meso-level dimension, referring to beliefs as well as actions in relation to relevant others where relational empowerment can be observed, and finally (3) the macro-level dimension, referring to outcomes in the broader, societal context where societal empowerment can be observed. In the context of women’s empowerment, capturing women’s self-confidence would be located at the micro level, women feeling and acting confident in relation to their partner or social network would be a meso-level outcome, and women’s situation in society would be located at the macro level.

Importantly, our aim is *not* to provide a full literature review, but an overview of different operationalizations of empowerment. We categorize different operationalizations into personal, relational, and societal empowerment to illustrate the importance of differentiating between these three dimensions. More precisely, we selected studies assessing commonly used quantitative and qualitative measures of women’s empowerment on the personal dimension, the relational dimension, and at a broader societal dimension (see **Table 1** for an overview of the discussed measures).

**Table 1:** Overview of reported operationalisations of women’s empowerment discussed in this article.

Dimension	Construct	Measures	Reference
Personal	Locus of control	A scale ranging from 0 (no control) to 3 (a strong personal control belief) was constructed by the sum of three items (adapted from Rotter, 1966). For each item participants were asked to choose between two options the one that best reflected their own belief. One option represented having control over life outcomes (e.g., ‘*what happens to me is my own doing*’) and one option representing having no control (e.g., ‘*sometimes I feel that I don’t have enough control over the direction my life is taking*’).	Morgan and Coombes, 2013; Hansen, 2015
	Self confidence	A scale was constructed based on a positive response to at least one of two questions. Specifically, participants were asked to indicate their confidence on a scale ranging from 1 (not at all) to 5 (very much). *How confident are you that you could raise your opinion in public?* And, *neighbors often share similar problems—how confident do you feel about offering advice to your neighbor?*	Burra et al., 2005; Kim et al., 2007
	Self-esteem	Self-esteem was assessed as one of the seven indicators of self-empowerment. Participants were asked to indicate on a scale from 1 (worse than before) to 5 (very good impact) the change they’d experienced since becoming a member of the MFI. *Self-esteem.*	Stromquist, 1995; Basargekar, 2009; Kato and Kratzer, 2013
	Self-efficacy	A scale ranging from 0 (no self-efficacy) to 3 (strong self-efficacy) was constructed by the sum of three scores. For each item participants were asked to indicate how many of the suggested actions they are comfortable doing. *Who do you interact freely with (tick as appropriate) (a) with own family members (b) with husband’s family (c) with neighbors (d) with personal friends outside family circle e) with local community leaders f) people in marketplace. At least four ticks = 1, otherwise = 0.*	Kato and Kratzer, 2013
Relational	Domestic violence	Data on violence was collected through structured interviews. Information on both physical violence (e.g., *slapping, beating, kicking, etc*.) and emotionally-abusive behavior (*e.g., not allowing the woman to visit her natal home*) was collected. Participants were asked to indicate whether any of the mentioned incidents had happened between herself and her husband in the preceding 4 months.	e.g., Goetz and Sen Gupta, 1996; Schuler et al., 1996; Rahman, 1999; Ahmed, 2005; Naved and Persson, 2005; Bali Swain and Wallentin, 2009
	Bargaining power	Bargaining power was assessed with 12 items assessing whether women were the primary decision-makers on 12 different expenditures or not. A distinction was made between total decisions (e.g., *food*), decisions on non-food expenditures (e.g., *home purchase and repair*), and decisions on loans (e.g., *investment*).	e.g., Duvendack et al., 2014; Banerjee et al., 2015; Datta, 2015
	Freedom of mobility	Participants were asked how they go to banks, markets, health centers, or places outside the village (except for their parents’ place). Participants were asked to choose one of the four answer options: does not go (=0), goes with husband or son (=1), goes with women (= 2), or goes alone (=3).	Pitt et al., 2006; Bali Swain and Wallentin, 2009; Datta, 2015
	Social network size	Participants were asked to indicate their social networks size by naming groups that they are an active member of (e.g., *MFIs; funeral associations; religious groups*).	Pitt et al., 2006; Sanyal, 2009; Hansen, 2015
	Social capital	Data on social capital was collected through semi-structured interviews. Participants were asked to reflect on any changes – before and after group membership – in four domains, such as *seeking and receiving help from others in times of personal and domestic crises.*	Sanyal, 2009
	Collective action involvement	Collective action involvement was assessed with four items assessing whether women engage in problem solving at the community level. Participants were asked to indicate whether they would act if she faces certain problems (e.g., *some women being beaten up, problems with the elected chief)*. Next, they were asked whether they would act by themselves, with other women, or not.	e.g., Kim et al., 2007; Sanyal, 2009; Datta, 2015
Societal	Percentage of female microfinance borrowers	Data for 435 microfinance institutions was obtained from MixMarket. The percentage of female borrowers was calculated based on the total loan portfolios of the microfinance institutions.	e.g., Hermes et al., 2011; D’Espallier et al., 2010
	Percentage of female borrowers with school-aged children in school	The percentage of female borrowers with school-aged children in school was calculated by dividing the number of female borrowers with school-aged children who state that all children are in school by the total number of female borrowers with school-aged children.	Women’s World Banking, 2013
	Percentage female leadership in MFIs	Data for 329 microfinance institutions was obtained from MixMarket. The percentage of female leadership in microfinance institutions was based on three categories for female leadership: *CEO, chair, and director.*	Strøm et al., 2014
	Percentage female staff promotion and attrition	The percentage female staff promotion and attrition was calculated by dividing the number of women voluntarily leaving the institution or the number of women promoted by the total number of women.	Women’s World Banking, 2013
	Average loan balance for female borrowers	The average loan balance for female borrowers was calculated by dividing female borrowers’ gross loan portfolio by the total number of female borrowers.	Women’s World Banking, 2013

*In the table above we report the dimension of women’s empowerment in the first column, in the second column we report the constructs used, in the third column we report one measure assessing this construct taken from the reference in bold, we added additional references in the fourth column.*

### Personal Empowerment

Previous research has assessed the impact of access to microfinance services on different components of women’s beliefs about their personal strength. Specifically, it has examined self-esteem (e.g., Stromquist, 1995; Basargekar, 2009; Kato and Kratzer, 2013), control beliefs (e.g., Morgan and Coombes, 2013; Hansen, 2015), self-confidence (Burra et al., 2005; Kim et al., 2007), and self-efficacy (e.g., Kato and Kratzer, 2013). We refer to these components as *personal empowerment* as they assess different psychological aspects about personal beliefs and actions. We have selected two different commonly used operationalizations, namely control beliefs (Hansen, 2015) and self-efficacy/self-esteem (Kato and Kratzer, 2013).

First, Hansen (2015) quantitatively examined the impact of a microfinance program (including skills training, saving activities, and micro loans) on psychological empowerment among women living below the poverty line in Sri Lanka. Women who had participated in the microfinance program for a period of 12–18 months were compared with a matched comparison group (no access to the program). To assess personal empowerment participants were asked to indicate their belief in their ability to control events affecting them with a self-report questionnaire (so called control beliefs, adopted from Rotter, 1966). Results indicated that women who had participated in the program reported higher levels of internal control beliefs compared to the comparison group.

Second, Kato and Kratzer (2013)^3^ quantitatively and qualitatively examined the impact of membership in microfinance institutions on women’s empowerment in Tanzania. Women who were members of the microfinance institutions were compared with non-members. Personal empowerment was measured with a self-report questionnaire assessing self-esteem and self-efficacy. Results indicated that women who were members of the microfinance institutions reported higher levels of self-esteem and self-efficacy than the comparison group. This result was further supported by in-depth interviews with ten members of the institutions who reported that participation in the microfinance program made them feel stronger and more respected by their families and community.

Further research in this field showed that women reported higher levels of self-esteem (e.g., Stromquist, 1995; Basargekar, 2009; Kato and Kratzer, 2013), stronger internal control beliefs (e.g., Morgan and Coombes, 2013; Hansen, 2015), and increased self-confidence (Burra et al., 2005; Kim et al., 2007). Overall, research investigating the impact of microfinance services showed mostly positive impacts for personal empowerment with respect to individual choice.

### Relational Empowerment

Other research on women’s empowerment has focused on women’s position in relation to relevant others, such as their partner, family, or social networks. Specifically, previous research examined the relation between access to microfinance services and women’s relationships with their partner by assessing women’s bargaining power within the household; the extent to which they have a say over household spending (e.g., Holvoet, 2005; Pitt et al., 2006; Duvendack et al., 2014; Upadhyay et al., 2014; Banerjee et al., 2015; Datta, 2015; Garikipati et al., 2016a), their freedom of mobility to visit places such as grocery stores or relatives outside the village (Pitt et al., 2006; Bali Swain and Wallentin, 2009; Datta, 2015) but also (risk of) intimate partner violence (e.g., Goetz and Sen Gupta, 1996; Kabeer, 1999; Rahman, 1999; Ahmed, 2005; Naved and Persson, 2005). Previous research also examined the relation between access to microfinance services and women’s membership in social groups (such as microfinance groups, school groups, religious groups, women’s groups) by measuring the number of social networks they are members of (e.g., Pitt et al., 2006; Sanyal, 2009; Hansen, 2015), seeking, receiving, or providing help in times of crises (e.g., Sanyal, 2009), or inclination to participate in collective action (e.g., Kim et al., 2007; Sanyal, 2009; Datta, 2015). We refer to these components as *relational empowerment* as they assess different aspects of women’s position in relation to others. Below we will illustrate three different studies, one investigating intra-household decision-making power (Banerjee et al., 2015), one investigating experiences of intimate partner violence (Rahman, 1999), and one investigating women’s social capital (Sanyal, 2009).

First, Banerjee et al. (2015) conducted a large-scale randomized control trial to investigate the impact of a group lending microcredit program on women’s intra-household decision-making power in India. Women who had received a micro loan through their participation in the microfinance program 15–18 months ago were compared with a control group (no access to the program). To assess relational empowerment participants were asked to indicate who takes decisions about spending money on twelve different expenditures (e.g., food, education, investment). These twelve indicators of women’s decision-making power were combined with four social indicators (e.g., number of female infants; enrollment of teenage girls) as a proxy for women’s empowerment. Results indicated that women who had participated in the program did not show an increase in women’s empowerment compared to the comparison group.

Second, Rahman (1999) set out to qualitatively examine the implications of a micro credit lending program in achieving equitable and sustainable development, including women’s empowerment in the context of a Grameen Bank in Bangladesh (participants could apply for a micro loan). To assess relational empowerment a variety of ethnographic methods were used to assess women’s experiences of intimate partner violence, and of violence by other members of the lending group and loan officers. Results indicated that a majority of female microfinance borrowers reported increased violence in the study village and increased violence and aggressive behavior (verbal aggression and physical assault) within the household because of their involvement with the bank.

Third, Sanyal (2009) conducted semi-structured in-depth interviews with female microfinance borrowers in India to examine the impact of microfinance services in promoting women’s social capital and their capacity to influence social norms and practices (participants received loans). Female borrowers who were a member of one of 59 microfinance groups were selected from a stratified random sample to participate in the research. To assess relational empowerment participants were asked about their levels of agency and of social capital before and after their group membership to generate retrospective data about changes in their ability to engage in actions that they could not perform before (e.g., ability to interact with people outside the family and kinship ties, physical mobility, participation in council meetings, seeking, receiving, or providing help in times of crises). The average period of group membership was 4 years. Results indicate that women’s membership in microfinance groups may improve their agency with respect to interpersonal behavior and facilitate social group membership.

Together, these studies suggest that microfinance services have mixed results regarding relational empowerment. Other research also showed mixed results, ranging from no effects to positive and even negative effects. For example, women, who participated in a microfinance program, showed no increase in intra-household decision-making power (e.g., number of expenditure decisions made by women; Banerjee et al., 2015), whereas another study indicated an increase in intra-household decision-making power (e.g., Pitt et al., 2006). Furthermore, some research provided evidence that women who received access to microfinance services experienced a decrease (e.g., Schuler et al., 1996; Kabeer, 1999; Copestake et al., 2001) whereas other research reported an increase in (risk of) intimate partner violence (e.g., Goetz and Sen Gupta, 1996; Rahman, 1999; Ahmed, 2005; Naved and Persson, 2005). Finally, research examining women’s engagement in social groups reported positive impacts such as larger social networks (e.g., Pitt et al., 2006; Sanyal, 2009; Hansen, 2015) and increased levels of seeking, receiving, or providing help in times of personal or domestic crises, as well as involvement in collective action (Sanyal, 2009). Overall, research investigating the impact of microfinance services showed mixed impacts for relational empowerment.

### Societal Empowerment

To the best of our knowledge, women’s empowerment in the societal dimension has so far been assessed with indices that map gender gaps in human development across nations such as the Gender Development Index or specific components such as the percentage of parliamentary seats held by women. In the context of microfinance, macro-economic analyses provide insights in for example the percentage of female microfinance borrowers (e.g., D’Espallier et al., 2010; Hermes et al., 2011), female clients with school aged children in school (e.g., Women’s World Banking, 2013), female leadership in microfinance institutes (e.g., Strøm et al., 2014), female staff promotion and attrition (Women’s World Banking, 2013), average loan balance for female borrowers, and financial literacy services offered to women (e.g., Women’s World Banking, 2013). Important to note, these studies focus on industry level indices of empowerment and do not relate to the societal level. In other words, they do not assess the impact of access to microfinance services on women’s empowerment in society but rather the impact of the mere presence of women in the context of microfinance services. We will illustrate this research with two different studies.

First, Hermes et al. (2011) examined the relationship between efficiency of microfinance institutions and outreach to the poor based on data from 435 microfinance institutes. Percentage of female microfinance borrowers was used as an indicator of outreach. On average across different loan types, 58% of the microfinance borrowers were female. The results indicate that there is a trade-off between outreach to women and efficiency of microfinance institutions. More precisely, the data suggests that microfinance institutes focusing more on female borrowers are less efficient with respect to financial performance by microfinance institutes. Second, D’Espallier et al. (2010) examined whether the percentage of female clients was related to repayment performance based on data from 350 microfinance institutions in 70 countries. The relationship between female clients and female gender bias in lending policies and indicators of repayment behavior (portfolio at risk, loan loss write-offs, and provisions) was examined. On average across different loan types, 73% of the microfinance borrowers were female. Results indicate that microfinance institutes with higher proportions of female borrowers have lower portfolio at risk and lower write-off rates indicating better repayment performance.

To conclude, these reported macro-economic effects of women’s empowerment offer insight in cross-country comparisons on gender performance of microfinance institutions and possible relations between gender performance and financial performance by the microfinance institutions. Previous research examining these relations shows mixed results. For example, the percentage of (poor) female microfinance borrowers was positively (e.g., Hulme and Mosley, 1996; D’Espallier et al., 2010; Quayes, 2015; Abdullah and Quayes, 2016), negatively (e.g., Cull et al., 2007; Hermes et al., 2011), or not (e.g., D’Espallier et al., 2010) related to increased financial performance by microfinance institutions. However, while the indicators used in this type of research (e.g., percentage female borrowers, percentage female staff) provide insight in the gender outreach and/or gender effectiveness of different microfinance institutions it does not highlight the position of the female microfinance borrowers themselves.

Thus, previous research in the field of microfinance has not yet operationalized women’s empowerment on the societal level as we suggest in this article. Using the mere presence of women in microfinance institutions as an indicator of women’s empowerment is too narrow (e.g., Geleta, 2013). The research mentioned above also illustrates the complexity and potential problems in grouping diverse groups of women together to investigate the outcomes of women’s empowerment. This approach may ultimately lead to a top–down way of discerning women’s empowerment. To gain a deeper understanding of women’s empowerment on the societal dimension, research should assess women’s position in society in two ways. Indeed, research should both examine women’s position by analyzing objective information about women’s social conditions (i.e., status) as well as, most importantly, examine women’s position relative to men (i.e., situation; for a similar argument see Johnston, 1985). We refer to women’s position at a broader societal dimension as *societal empowerment*. Thus, we suggest that future research should follow female microfinance borrowers over time to investigate how they achieve more opportunities and rights (e.g., voting; Johnston, 1985; Beteta, 2006; education; Dijkstra, 2002). Additionally, future research should investigate how women can use these gains effectively to improve women’s interests at large. For example, by striving toward improvements in women’s position for future generations, such as more strongly supporting their daughters to successfully attend schooling (e.g., Kabeer, 1999; Banerjee et al., 2015), and follow different career trajectories.

## Three-Dimensional Women’S Empowerment Model

We offer a framework suggesting that women’s empowerment can occur at three distinct but related dimensions: the personal, relational, and societal dimension. Based on our review of previous research we find different effects of access to microfinance for each of the three dimensions of women’s empowerment. With the risk of oversimplifying this complex matter, we suggest that the review shows first, that access to microfinance services was associated with higher levels of *personal empowerment*, such as increased personal control beliefs (e.g., Hansen, 2015). Second, female microfinance borrowers showed higher levels of *relational empowerment* on the level of social group memberships, such as larger social networks (e.g., Pitt et al., 2006). However, on the level of intimate relationships we found mixed results, showing for example both increased as well as decreased decision-making power by female borrowers (e.g., Banerjee et al., 2015). Third, with respect to *societal empowerment*, a positive signal is that the percentage of female borrowers receiving microfinance services is relatively high; but research provided mixed results about women’s financial performance, showing positive as well as negative relations between outreach to female borrowers and financial performance by microfinance institutions (e.g., Hermes et al., 2011). Important to note, research so far has not tapped into our understanding of societal empowerment as women’s situation relative to men in a broader societal dimension.

Our Three-Dimensional Women’s Empowerment Model borrows the assumption from the ecological systems theory (Bronfenbrenner, 1994) that people do not exist in a social vacuum but encounter different environments throughout their life that may influence their behavior. The ecological system theory, focusing on the development of children, proposes that individuals directly influence their own experiences and vice versa within specific microsystems (e.g., family, school) and between different microsystems (mesosystems). People’s development can also be influenced by settings that the individual is not directly part of (i.e., exosystem: e.g., schoolpolicy). Bronfenbrenner (1994) argues that these three lower-order systems combined, constitute consistencies fitting with relevant cultural ideologies. Next, this cultural macrosystem is influenced by time, such that the past influences the present. Our Three-Dimensional Model of Women’s Empowerment broadly adheres to the same general structure and underscores the importance of the interplay between individuals and their environment.

Additionally, our proposed three-dimensional model concurs with other research noting the importance of considering changes at the individual, the relational, and the communal level when examining processes related to social change for women (e.g., Kabeer, 1999; Grabe, 2012). Importantly, our model closely ties into the empowerment process described by Rowlands (1997) in the context of social work and education. Rowlands stressed that women’s empowerment occurs at three levels – the personal, close relationships, and collective – and that these three levels have to be taken into account simultaneously when trying to investigate empowerment. We agree with Rowlands’ claim and propose that full women’s empowerment entails all three dimensions of empowerment. However, different from Rowlands we suggest that it is possible to promote and examine empowerment at each dimensions of empowerment independently, depending on one’s research focus and the context in which it is embedded. In fact, we stress that women’s empowerment effects on multiple dimensions need to be differentiated and not combined. While it is common practice in program evaluations to use women’s empowerment indices that aggregate result from several indicators across key areas (e.g., Women’s empowerment in Agriculture Index, Alkire et al., 2013), we fear that these aggregates don’t do justice to the different dimensions at which empowerment can be observed.

Most importantly, we stress that one should *clearly specify on which dimension of empowerment an intervention focuses* to offer more systematic insights in women’s empowerment across studies. If research would only focus on the personal dimension of women’s empowerment (e.g., self-esteem, personal control beliefs) and use these insights to directly conclude that access to microfinance services strengthens women’s empowerment within her social environment, this could provide a skewed insight and may have undesired policy implications. More specifically, when operationalizing women’s empowerment in terms of women’s personal control beliefs it is possible that women feel personally more in control (‘I know what I am doing’), but not in relation to their partner (‘My partner gets aggressive if I try to have a say in important decision-making’). In fact, previous research suggests that women’s increased autonomy resulting from her participation in microfinance services can destabilize the relationship between the female microfinance borrower and her husband and thereby increase the risk of intimate partner violence (e.g., Goetz and Sen Gupta, 1996). This may explain the mixed results presented at different dimensions of women’s empowerment (i.e., personal and relational) and illustrates the importance of carefully and explicitly choosing different aspects of women’s empowerment and defining at which dimension(s) an intervention may have impacts. In the following, we discuss two aspects that influence the development of women’s empowerment, namely time and culture.

### The Role of Time in Women’s Empowerment

Women’s empowerment is seen as a process rather than a fixed outcome (e.g., Bandura, 1986; Kabeer, 1999; Malhotra et al., 2002; Maton, 2008) and described as the development from being un-empowered to becoming empowered (e.g., Kabeer, 1999; Bali Swain and Wallentin, 2009). As such, already the definition of women’s empowerment underscores the importance of time in understanding its development. However, we know surprisingly little on how women’s empowerment may develop over time. The proposed Three-Dimensional Women’s Empowerment Model may deepen our understanding of the development of women’s empowerment by disentangling the different dimensions where empowerment can be observed. However, we can only speculate about the order in which the three dimensions might develop. Moreover, we stress that the relation between access to interventions and the development of women’s empowerment on the personal, relational, and societal dimension may be time-dependent.

First, if we consider the example of training offered in the context of microfinance services and thus the bottom-up development of women’s empowerment, we may expect personal empowerment to develop within a relatively short time-span. Training in itself may increase people’s self-efficacy and control beliefs, because people can experience their ability to perform certain tasks and increase their beliefs in their capabilities through training (Bandura, 1997). Yet, changing relational dynamics may take more time (e.g., Inglehart and Norris, 2003). Empowerment on this dimension is dependent upon other actors and may require more structural transformations (e.g., Dixon et al., 2012). Therefore, we suggest to only consider any impact of interventions on relational empowerment over a longer time-span of at least a few years. Lastly, societal empowerment is not likely to be instigated by any single intervention as it is highly related to cultural norms and traditions. Nonetheless, we suggest that societal empowerment could possibly develop over time, though it may be that this dimension of empowerment can only be observed after years (e.g., the new generation), which makes it complex to draw any conclusions about directionality or even causality. Thus, we expect that time may determine whether or not any result can be expected and observed on each of the three dimensions of women’s empowerment. Also, other research argued that the time path of a program should be considered in the timing of evaluations (e.g., King and Behrman, 2009; Bonilla et al., 2017). To better understand whether effects take time to materialize or whether effects that emerge quickly persist one should measure outcomes longitudinally (McKenzie and Woodruff, 2014).

Second, we propose that the three dimensions are related but that the directionality of the model is not fixed. Even though some sequences may be more probable then other, we stress that women’s empowerment can be instigated at any of the three dimensions or at multiple dimensions simultaneously. In the context of microfinance services, we suggest that women’s empowerment may be a bottom–up process instigated on the personal dimension (i.e., through increased personal agency by contributing to the household income), which may then instigate the experience of empowerment on the relational and/or societal dimension. In line with this suggestion, previous psychological research conducted in the context of microfinance services stressed that women should first become aware of the options that they are individually capable of taking – i.e., their personal capacity – before they can actually proceed to influence aspects that are important to them in their daily life (Hansen, 2015). Similarly, political scientists examining the cross-cultural development of gender equity argue that women must experience personal change before relational power distributions can change (Inglehart and Norris, 2003).

In the context of microfinance services, women’s empowerment may thus be seen as a process typically starting with personal empowerment and resulting in empowerment at all three dimensions, with societal empowerment as the final aspect to develop (for a similar argument see Kabeer, 2005). We recognize that this proposed sequence between personal and relational dimensions is based upon an understanding of individuals as independent agents of choice. However, women’s empowerment might also be instigated on the relational dimension (i.e., small collectives; Stromquist, 1995). Nonetheless we expect societal empowerment to develop last because societal power is deeply rooted in social systems and values. It is therefore unlikely that any single intervention will completely alter power and gender relations (e.g., Cheston and Kuhn, 2001). Other authors similarly argue that gender inequity within societies may ensure that increased intra-household decision-making power (relational empowerment) will not result in structural societal changes (e.g., Johnson, 2005; Guérin et al., 2015). However, the changes instigated on the personal and relational dimension through access to microfinance services might over time also contribute to women’s empowerment on the societal dimension. Empowerment on the societal dimension may then best be compared with gradual social change where cultural characteristics such as norms and values change (Pinquart and Silbereisen, 2004; de la Sablonniere, 2017), which can bring about both cultural gains (i.e., more gender equity) and losses (i.e., less social belonging; Greenfield, 2016).

Importantly, such bottom–up development of women’s empowerment is not the only option. One example for a top–down approach to stimulate women’s empowerment starting on the societal dimension is setting gender quotas (e.g., percentage of leadership positions reserved for women). Such an approach in politics aims to increase women’s presence in legislature and to improve gender-related policy outcomes such as inheritance rights (e.g., Htun and Jones, 2002). This example illustrates one other possible direction in the process of women’s empowerment in which an intervention is implemented at the societal level and should result in empowerment in the other two dimensions.

In sum, we suggest that time is crucial in predicting empowerment effects. First, the model suggests that the time lag between an intervention and its evaluation may influence when empowerment effects on the different dimensions are likely to be found Second, the model suggests that the three dimensions are related but that the sequence in which they can be observed depends on the implemented type of intervention.

### The Role of Culture in Women’s Empowerment

In the current article we discussed studies conducted in a variety of different cultural contexts, such as Sri Lanka, Bangladesh, and Tanzania. Obviously, there are important differences between these cultures. Culture can be defined as the dynamic patterns of ideas, practices, institutions, products, and artifacts that are shared by certain groups of people (Markus and Kitayama, 2010). While individual differences between people from the same cultural background are omnipresent, people within the same culture tend to hold similar values, beliefs, and practices (e.g., Smith et al., 2013). Across cultures, people may thus for example differ in how they construe their self-concept (independent or interdependent; Markus and Kitayama, 1991), to what extent they tolerate deviant behavior, and how strongly they adhere to social norms (tight or loose cultures; Gelfand et al., 2011). It may be crucial to consider these social norms in understanding and stimulating social change (Tankard and Paluck, 2016).

As highlighted in previous research, gender relations vary both geographically and over time and therefore should always be investigated in specific contexts and pertain to realities of women’s lives rather than being based on a generalized assumption that they are oppressed (Mosedale, 2005; Haase, 2011; Kurtiş and Adams, 2015). Indeed, due to the diversity in interventions and cultural differences, access to microfinance cannot be expected to have one single consistent impact story (Garikipati et al., 2016b). Instead, previous research underscores the importance of considering factors such as cultural norms and attitudes in the development of women’s empowerment (e.g., Johnston, 1985; Mayoux, 1999; Armendáriz and Morduch, 2010; Sardenberg, 2010). In fact, it has been stressed that empowerment develops through the interaction between the individual and the cultural context (e.g., Narayan, 2005) and that failure to consider socio-political and cultural structures can reinforce existing power imbalances (e.g., Dutt et al., 2016). Below we discuss how culture influences the meaning of women’s empowerment.

First, previous research suggests that often-used indicators of women’s empowerment reflect an understanding of women’s empowerment based on culturally specific practices (e.g., female seclusion in South Asia) that may not apply to other cultures (e.g., Heckert and Fabric, 2013; Duvendack and Palmer-Jones, 2017). In line with this assumption, qualitative research conducted in Guatemala concluded that local women from five communities in Chimaltenango and Quetzaltenango did not feel empowered by having sole autonomy and decision-making power within the household but rather sought the involvement of their husbands (Carter, 2002). A similar conclusion was drawn based on narratives of Bangladeshi and Afghan women who chose quite different pathways of change, shaped by culturally unique norms, values and institutions, in seeking a greater degree of agency in their own lives (Kabeer, 2012). While for the interviewed Afghan women awareness of different realities experienced through migration and different regimes influenced personal empowerment, for the interviewed Bangladeshi women personal empowerment translated into greater awareness of rights and willingness to fight for them on a societal level. Moreover, how people experience each of the three dimensions of empowerment may differ based on diverse understandings of the self and the society across cultures. In cultural contexts where the social world is perceived as a dense network of connections, characterized by obligations for care and support (Kurtiş et al., 2016), women’s experience of personal empowerment may be more relational than in cultural contexts where the social world is perceived as more independent. For example, research examining the impact of women’s business ownership on women’s empowerment among Maasai women in Tanzania showed that cooperative business ownership was more strongly related to women’s empowerment than individual business ownership (Dutt et al., 2016). The authors suggest that the cooperative business ownership was more successful because it adhered to local cultural norms of social relations by emphasizing the community rather than the individual (Dutt et al., 2016; Kurtiş et al., 2016).

Indeed, psychological scholars highlight the necessity to draw upon local understandings to resonate with local realities and better serve local communities (Adams et al., 2015). Since women in local communities are best aware of what women’s empowerment means to them, it may thus be crucial to allow them to set their own agenda in matters related to enhancing their own sense of empowerment (Stromquist, 1995; Kurtiş et al., 2016). Hence, members of local communities should be involved to facilitate culturally relevant social change without marginalizing women’s voices (Dutt et al., 2016). While the potential lack of generalizability and tendency to overlook problematic indigenous practices may need to be considered (Adams et al., 2015), this strategy allows us to not only offer culturally adapted interventions but also reconsider often-used concepts (e.g., Comaroff and Comaroff, 2012). As argued in previous research, access to microfinance services may only empower women if cultural norms and expectations are taken into account (e.g., Geleta, 2013). In line with this theorizing, we expect that cultures influence how women’s empowerment is defined, which aspects are important, and which components reflect women’s empowerment on each of the three dimensions. Accordingly, we expect that one intervention can have diverse impacts on each of the three dimensions of women’s empowerment in different cultural contexts. For example, an intervention through which women gain more economic independence might only increase women’s likelihood of leaving their partner in societies where divorced women are not seen as social outcasts.

Second, women’s empowerment is considered as a process wherein women challenge existing norms and culture of the society in which they live (Bali Swain and Wallentin, 2009). Accordingly, it is crucial to be aware of the cultural context and the position of women in it. Previous research highlighted that culturally defined norms and practices should be considered for a transition away from classic patriarchy to develop (Kandiyoti, 1988). Some form of patriarchy is prevalent across almost all cultures (e.g., Stockard and Johnson, 1992). However, psychological research indicated that cultures differ in the extent to which they value gender equity (e.g., Hofstede et al., 2010) and the extent to which certain gender roles are subscribed to (e.g., McCrae et al., 2005). Importantly, these gendered norms and beliefs may mediate the relation between structural equity and female suppression (Archer, 2006). Indeed, previous research reported a link between adhering to patriarchal values and sexual violence against women (e.g., Yodanis, 2004). In countries where women held a weaker position in society men more frequently showed physical aggression toward women relative to the frequency with which women showed physical aggression toward men (Archer, 2006).

In sum, the prevalence of gender inequity may obstruct possible structural societal changes resulting from access to microfinance services (e.g., Guérin et al., 2015). Empirical evidence supports this assumption. Indeed previous research analyzing the impact of fifteen different programs in Africa reports that women’s empowerment depends on inflexible, household- and region-specific, social norms, and traditions (Mayoux, 1999). Similar conclusions were drawn based on a five-country study in Asia, which indicated that gender norms strongly influence the extent to which women experience empowerment (Oppenheim Mason and Smith, 2003). Thus, we propose that it is important to understand the cultural context and the position of women in society to understand the development of women’s empowerment.

To conclude, we suggest that cultures may differ with respect to which components of empowerment are appropriate indicators of empowerment. Moreover, we suggest that the cultural context should be considered to properly understand the development of women’s empowerment. Accordingly, when developing interventions, cultural norms should be identified and described when presenting impacts, thereby facilitating comparison between studies. To investigate at what time access to an intervention impacts women’s empowerment at each of the three different dimensions across cultures, we encourage future longitudinal and cross-cultural research to examine the development of women’s empowerment on the personal, relational, and societal dimension.

## Implications and Future Perspectives: Toward a Better Understanding of Women’S Empowerment

In this paper, we aimed to increase our understanding of women’s empowerment and how it should be studied in future research. We can derive four main conclusions based on our work: First, women’s empowerment might best be conceptualized as a multifaceted process, which involves individual as well as collective awareness, beliefs, and behavior embedded in the social structure of specific cultural contexts. Second, based on the research reported above examining the impact of access to microfinance services on the development of women’s empowerment, we concur with conclusions by previous research (e.g., Duvendack et al., 2014; Vaessen et al., 2014) that inconclusive results exist on the relation between microfinance and women’s empowerment. Previous research has suggested that existing misconceptions over the potential gender effects of microfinance stem from a simplistic vision of the complex process that is empowerment (e.g., Garikipati et al., 2016b). This is in line with our third conclusion: the impact of access to microfinance services on the development of women’s empowerment is hard to assess, because it is difficult to properly compare results across studies. However, if we differentiate between the three dimensions of empowerment specified in the Three-Dimensional Model of Women’s Empowerment such comparisons may be improved and more consistent patterns of findings may emerge. Fourth, two crucial moderators of women’s empowerment, time and culture, should be considered to increase our understanding of women’s empowerment and its development.

Most of the work discussed in this paper operationalized empowerment based on an understanding of women as individual agents of change. However, including empowerment measures acknowledging the importance of vicarious others in women’s experiences of empowerment – focusing on the beliefs others in one’s network hold about an individual versus own beliefs – may enrich our understanding of women’s empowerment. Thus, concurring with the decolonial feminist perspective (e.g., Kurtiş and Adams, 2015) we suggest that future research should be sensitive to cultural contexts, and gain insights from the experience of women in majority-world spaces. We invite future research to develop measures to assess women’s empowerment based on local operationalizations and different perspectives. Moreover, we propose that by focusing on three dimensions of empowerment, our model offers one way to consider the relativity of context and culture in women’s empowerment. By considering not only on individual dimensions of empowerment but also on relational and societal empowerment we provide a first suggestion toward an understanding of women’s empowerment that also applies to cultural worlds of embedded interdependence (see Markus et al., 1997).

Importantly, we have focused on the measurement of women’s empowerment in the context of microfinance services. As such, the proposed model is most strongly substantiated in this specific context. Nonetheless, we propose that the suggested differentiation between three different dimensions may also apply to different interventions, which aim to strengthen the position of women. Additionally, in accordance with previous work (e.g., Kurtiş and Adams, 2015) we propose that the need for empowerment exists across the globe and is not unique to majority-world spaces. While most of the cited research was conducted in these societies we suggest that the different dimensions of empowerment are similarly applicable to women in WEIRD (Henrich et al., 2010) settings. Additionally, just as women’s empowerment can be analyzed on personal, relational, and societal dimensions, this should similarly apply to other forms of empowerment for different marginalized groups. For example, we propose that this framework could also be used to understand the impact of diversity and inclusion-programs in industry-settings (e.g., International Labour Organization, 2014). We invite future research to use this general framework in different contexts and among different target groups.

We derive two main implications from our work. First, we suggest that future research should differentiate between the three dimensions of women’s empowerment specified in the Three-Dimensional Model of Women’s Empowerment, thereby increasing our understanding of women’s empowerment and its development and facilitating comparison of results between studies and cultures. We hope that our model encourages future research to focus more on the development of women’s empowerment over time. As a result, stronger theories may develop regarding how and why certain components on each dimension of empowerment could be impacted by different interventions. Second, but related, we suggest that program designers should specify how an intervention should stimulate which dimension(s) of women’s empowerment. When developing a theory of change (White, 2009), detailing how and why activities will bring about anticipated changes in the short- and in the long-term, program designers should consider the three dimensions of women’s empowerment. Moreover, researchers and program designers should consider after what time they would expect specific impacts on each of the three dimensions of women’s empowerment in specific cultural contexts. We propose that the choice of intervention and of cultural context has consequences for to be expected pathway through which women’s empowerment may develop and be observed.

## Conclusion

Empowering women is seen as one of the central issues in the process of sustainable development for many nations worldwide (e.g., Sen, 1999; Organization for Economic Cooperation and Development [OECD], 2012; United Nations Economic Commision for Europe [UNECE], 2012; Gates, 2015). Around the globe, governments and different organizations strive to increase women’s empowerment by implementing different interventions such as offering access to microfinance services to promote sustainable development and human rights.

The Three-Dimensional Model of Women’s Empowerment integrates different literatures studying the impact of offering microfinance services on women’s empowerment. The core premise of the model is to differentiate between three different dimensions of women’s empowerment, namely (1) personal empowerment, referring to individual’s personal beliefs as well as actions, (2) relational empowerment, referring to beliefs as well as actions in relation to relevant others, and (3) societal empowerment, referring to the situation of women in the broader societal context to understand how women’s empowerment may develop. Furthermore, unraveling two important moderators of empowerment, namely time and culture, the model allows a more dynamic understanding of why some women may feel more empowered than others, why some women may express higher levels of personal but not relational empowerment, and why one specific microfinance intervention may show positive impacts on women’s empowerment in one but not another nation. Integrating all three dimensions of women’s empowerment into one research model provides new theoretical insights into how women’s empowerment may develop through access to microfinance services and offers clear practical implications for involved stakeholders in the field.

## Author Contributions

MH, NH, SO, and RL contributed to the discussion and development of the conceptual framework of this article. MH and NH conducted the literature review, analyzed the results, and wrote the manuscript. SO contributed to the rewriting of the manuscript. All authors approved the final version of the manuscript.

## Conflict of Interest Statement

The authors declare that the research was conducted in the absence of any commercial or financial relationships that could be construed as a potential conflict of interest.

## Footnotes

1 We focus on the different dimensions on which women’s empowerment can be measured, not the different levels on which interventions can operate. However, we note that microfinance initiatives targeting individual women compared to initiatives targeting women’s groups may yield different outcomes in terms of women’s empowerment across all three dimensions (e.g., Dutt et al., 2016).

2 The validity of some of the different measurement-techniques has been debated (e.g., self- report; Cook and Campbell, 1979). However, in the current paper we aim to offer an overview of the different measures used to operationalize dimensions of women’s empowerment and will thus not engage in this debate.

3 For this article we have focused on two measures reported in this article representing personal empowerment, namely self-esteem and self-efficacy.
